# Comment on “Fatigability: A Prognostic Indicator of Phenotypic Aging”

**DOI:** 10.1093/gerona/glab059

**Published:** 2021-02-27

**Authors:** Lotta Palmberg, Erja Portegijs, Laura Karavirta, Taina Rantanen

**Affiliations:** Gerontology Research Center and Faculty of Sport and Health Sciences, University of Jyvaskyla, Finland

Increasing research interest in fatigability has resulted in increased efforts targeted toward its assessment. We have read with great interest the article by Schrack et al. (1). The authors have made a valuable contribution to advance fatigability research among older adults by reviewing existing literature and frequently used measures. Fatigability has been divided into 2 dimensions; perceived fatigability and performance fatigability. The latter is characterized by decline in performance during a standardized task (2). Thus, people with higher performance fatigability will exhibit greater decline during tasks standardized to a certain demand level (eg, walking speed) than people with lower performance fatigability.

When assessing performance fatigability, there are some instances where a self-paced walking test is preferred, as it may better reflect daily life situations (3), especially among older people. We wish to propose an alternative computation method of performance fatigability during a self-paced 6-minute walk test (6MWT) to those mentioned in the article. Our method also aims to overcome some concerns that we perceive related to 2 measures utilizing self-selected pace of walking (3,4).

To clarify the concern that we have over these measures, we used the equation by Murphy et al. (4) as an example. The equation for computing performance fatigability based on 6MWT was described as follows:


$$\begin{array}{ll} Fatigability\;1 & =\;\frac{a}{b}*1,000\\ & =\frac{\left(\frac{MWS\;\;\;over\;\;\;6\;minutes\;\;\;}{MWS\;\;\;over\;\;\;f\;irst\;\;\;2\;minutes}\right)}{Total\;\;\;distance\;\;\;walked}*1,000\; \end{array}$$

First, *a*, the ratio of average walking speed (MWS, m/s) relative to the beginning, is calculated. Then, to account for task demand, *a* is divided by total distance (m) walked during the test (*b*), and, to obtain meaningful scores, multiplied by 1,000. Authors report that higher scores indicate higher performance fatigability.

However, in line with the definition of performance fatigability, those experiencing largest *decline* in walking speed and the lowest overall walking speed would be expected to get highest total scores. To our best understanding, the above-mentioned equation produces highest scores for those walking generally at a slower pace (low *b*) but who *increase* their walking speed toward the end (high *a*). A greater slowing during the test results in lower scores, as ratio *a* decreases (*a* < 1.0) compared to having stable (*a* = 1.0) or increasing walking speed (*a* > 1.0). Those with overall slower walking speed get higher scores than faster walkers, as *b* decreases. Therefore, the measure seems to identify those walking slowly rather than higher performance fatigability per se.

We propose a modified computation method to overcome the limitation described above, and conducted an initial validation for this new equation.

We computed performance fatigability scores based on data from a self-paced 6MWT and used the ratio of change in lap times (s) rather than in walking speed (m/s) in the equation. We used lap times of the second (beginning) and second-to-last lap (end), based on the approach by Simonsick et al. (5).


$$\begin{array}{ll} Fatigability\;2\; & =\;\;\frac{a}{b}\;*\;1,000\;\\ & =\;\frac{\left(\frac{Lap\;\;\;time\;\;\;end}{Lap\;\;\;time\;\;\;beginning}\right)}{Total\;\;\;distance\;\;\;walked}\;*\;1,000\;\;\; \end{array}$$

Highest scores are obtained by those slowing their walking during the test (*a* > 1.0) and having lower overall walking speed (low *b*). Thus, higher scores indicate higher performance fatigability in line with its definition.

We used data from a population-based sample of 778 Finnish community-dwelling 75-, 80- and 85-year-olds participating in AGNES study (6). For the 6MWT, participants walked 40-m laps at their usual pace in an indoor corridor. Study measures included health, function, and physical activity, and alternative measures of fatigability. Fatigability measures were a modified perceived exertion fatigability (PEF) during the 6MWT (4), and self-reports of the Physical Fatigue Subscale (PFS) and total score of the Situational Fatigue Scale (SFS). Other measures were usual 10-m gait speed, Short Physical Performance Battery, self-reported walking difficulty over 500 m, Yale Physical Activity Survey, age, and chronic conditions.

Correlations were tested with Spearman’s rho. Our performance fatigability score showed a relatively strong correlation with PEF (rho 0.67) and moderate correlations with SFS (0.42) and PFS (0.49; Table 1). Correlations with other measures were in expected directions, and particularly strong for 10-m gait speed (0.79).

**Table 1.** Spearman Correlations With Other Fatigability Measures, and Measures of Health, Function, and Physical Activity, *n* = 778

	PEF	SFS	PFS	WD 500 m	10-m Gait Speed	SPPB	YPAS	Age	Chronic Conditions
Performance fatigability	0.67	0.42	0.49	0.46	−0.79	−0.56	−0.41	0.31	0.30

*Note*: PEF = perceived exertion fatigability during the 6-min walk test; PFS = Physical Fatigue Subscale of the Situational Fatigue Scale; SFS = total score of the Situational Fatigue Scale; SPPB = Short Physical Performance Battery; WD = self-reported walking difficulty over 500 m; YPAS = Yale Physical Activity Survey. *p* < .001 for all.

Theoretically, *Fatigability 2* fits better with the definition of performance fatigability than the earlier computation methods. An additional advantage is that fewer conversion steps are needed for the equation (ie, lap times are not converted to walking speed, or averages calculated). The initial validation reported here is promising, but more research is warranted. For example, more information is needed for optimal use of task demand in standardizing performance fatigability score when using self-paced walking tests.

## Funding

The research was funded by Academy of Finland (310526 to T.R.) and the European Research Council (693045 to T.R.).

## Conflict of Interest

None declared.
